# Dissociation of Implicit and Explicit Interpretation Bias: The Role of Depressive Symptoms and Negative Cognitive Schemata

**DOI:** 10.3390/brainsci13121620

**Published:** 2023-11-22

**Authors:** Michèle Wessa, Mila Domke-Wolf, Stefanie M. Jungmann

**Affiliations:** 1 Department of Clinical Psychology and Neuropsychology, Institute for Psychology, Johannes Gutenberg-University Mainz, 55122 Mainz, Germany; 2 Research Group Wessa, Leibniz-Institute of Resilience Research (LIR), 55122 Mainz, Germany; 3 Department of Clinical Psychology, Experimental Psychopathology and Psychotherapy, Institute for Psychology, Johannes Gutenberg-University Mainz, 55122 Mainz, Germany; jungmann@uni-mainz.de

**Keywords:** ambiguous cue conditioning task, dysfunctional attitudes, ambiguous social scenarios task

## Abstract

A negative interpretation bias appears to depend on several depression-related state and trait characteristics, most notably depressive symptoms, negative mood, and negative cognitive schemas. While empirical findings for explicitly assessed interpretation bias are rather consistent, implicit measures have revealed heterogeneous results. In this context, we present two studies investigating the relationship between implicit and explicit interpretation bias and depression- and anxiety-related state and trait variables. In the first study, we conducted an implicit ambiguous cue-conditioning task (ACCT) with 113 young, healthy individuals. In the second study, we utilized an explicit ambiguous social situations task (DUCTUS) with 113 young, healthy individuals. Additionally, a subsample of 46 participants completed both the ACCT and DUCTUS tasks to directly relate the two bias scores obtained from the implicit and explicit assessment methods, respectively. In the first study, regression analysis revealed no significant predictors for the implicit interpretation bias. However, in the second study, the explicit negative interpretation bias was significantly predicted by female gender, depressive symptoms, and dysfunctional cognitive schemas. For the subsample that completed both tasks, we observed no significant correlation between the two bias scores obtained from the ACCT and DUCTUS. These results suggest that implicit and explicit interpretation biases are differently associated with depression-related trait and state characteristics, indicating that they represent different aspects of biased information processing.

## 1. Introduction

The way we think about an event and how we evaluate it influences our immediate emotional response to this event (e.g., [1,2]). Thus, consistently interpreting an ambiguous situation (or internal state) in a positive or negative manner has long-term consequences on an individual’s mood and behavior. As such, a negative interpretation bias, referring to the systematic selection of negative or threatening interpretations when resolving ambiguous situations, has been not only suggested to play a crucial role in the etiology of depression (e.g., [3,4]), but has also been consistently associated with depressive symptoms, clinical depression (e.g., [5,6,7]), and anxiety disorders, as well as aggression and substance abuse [8,9]. Furthermore, the absence or reduction of a positive interpretation bias has been linked to depressive symptoms in adolescents at risk for developing depression [10]. Based on this consistent empirical evidence, intervention methods aiming at the modification of such biases have been developed in recent years [11,12]. Cognitive bias modification training significantly reduces attentional as well as interpretation biases in depression [13,14], as confirmed by a recent meta-analysis [15].

The biased interpretation of an ambiguous situation in a negative manner appears to depend on various individual and situational factors. At the individual level, these factors can include trait characteristics, such as general beliefs and schemas, as well as trait anxiety. They also encompass state characteristics, such as depressive symptoms, positive or negative mood, and state anxiety [3,7,16,17].

Cognitive schemata, referring to a network of mental representations that individuals use to organize and interpret information from their environment, play a particularly crucial role in generating emotions because they can be seen as a mental framework that influences the attention, perception, encoding, selection and retrieval of information, and subsequent decision-making [3]. Beck and colleagues [18] postulated that cognitive schemata are shaped by previous experiences and are essential in guiding rapid decision-making in ambiguous and unclear situations. According to Ellis [19], irrational beliefs, in particular, lead to negative emotional states, and Lazarus [20] proposed that these irrational beliefs create a general vulnerability and biased information processing that occurs automatically, at least partly unconsciously. As a result, cognitive schemata can trigger a mood-congruent interpretation and appraisal of a situation as positive or negative, which, as described earlier, can elicit a corresponding emotional response [1,3]. When exposed to a vicious circle of negative interpretation and appraisal of situations, corresponding negative emotional responses reinforce the negative scheme. In turn, this can lead to the development of depressive or other psychopathological symptoms [16]. In that regard, negative interpretation biases can significantly disrupt the trajectory of emotion generation by initiating an inflexible, automatic, and mostly unconscious process of early negative appraisal. Difficulties in discarding initial negative interpretations of a given situation may result in recurrent ruminative thoughts in depression [21], which, in turn, can foster mood-congruent appraisal processes [14]. Indeed, Kuehner and Huffziger [22] demonstrated that inducing a ruminative self-focus after negative mood induction significantly increased dysfunctional attitudes in healthy study participants.

When reviewing the literature on interpretation biases in various psychopathologies and non-clinical samples, as well as cognitive bias modification training (with a focus on interpretation), it becomes apparent that there is a wide range of methods used to assess (or train) interpretation biases, and the results show significant heterogeneity on the methods employed [7,13]. Most studies have utilized explicit (also known as offline) tasks to measure interpretation bias. These tasks include the word association task, the sentence completion or scrambled sentence task, and the ambiguous scenario task (for a comprehensive overview of various offline tasks and their strengths and weaknesses, see [14]). These methods are relatively straightforward to administer and significantly contribute to our understanding of biased information processing. They often incorporate salient stimulus material that is self-relevant to the participants, such as scenarios involving success or failure, disappointment, and daily life experiences with friends or work colleagues. However, it is important to know that in these tasks, the response to an ambiguous stimulus often occurs after reflecting rather than immediately following the stimulus, making these tasks more susceptible to several biases [14]. First, demand effects may impact the results of these measures, meaning that study participants respond in a manner that aligns with the investigator’s assumed expectations. Second, a selection bias could be present, with participants choosing from a range of interpretations or evaluations instead of providing an immediate spontaneous conclusion. Third, a reporting bias might influence the results when participants follow their general tendency to report more negative interpretations, even if their actual interpretation is not negative [23]. To mitigate these potential caveats, implicit (also known as online) assessment methods can be employed, in which ambiguity is resolved at that moment the ambiguous stimulus is encountered. Such methods may involve response latencies [24], startle reflex amplitudes [25], or response choices after a conditioning procedure [26,27]. Results from these implicit assessment methods of interpretation bias have yielded heterogeneous findings, with some methods showing significant associations between a negative bias and depression [24,28], whereas others have not [25]. It is worth noting that negative interpretation biases have been consistently observed in patients with unipolar depression when assessed with explicit tasks but not when obtained from implicit tasks [7].

To further elucidate the differential contributions of depression- and anxiety-related state and trait variables to explicitly (offline) and implicitly (online) measured interpretation biases, we conducted two studies: The first study employed the Ambiguous Cue Conditioning Task (ACCT; Study 1) as an implicit (online) measure of a negative interpretation bias. This task was adapted from animal research [27,29,30], where an affect-related bias in interpreting ambiguous cues after mood manipulation (anxiety, depression) has been consistently observed across species [31,32,33]. The second study employed an explicit (offline) measure of a negative interpretation bias, the Dysfunctional Cognitive Thoughts Stories (DUCTUS; Study 2), adopting the idea of an ambiguous social situation task that was initially used in cognitive bias modification training [34]. Participants were asked to choose one of three alternatives for how an initially ambiguous situation would end (either positive, negative, or neutral), reflecting their interpretation (endings based on Beck’s cognitive theory [18]).

To evaluate whether previously observed heterogeneous findings might result from the fact that different assessment methods, such as an explicit evaluation task or an implicit conditioning task, measure different aspects of the biased interpretation, we investigated the impact of depression-related predictors (depressive symptoms, negative mood, and dysfunctional cognitive schemata) on implicitly and explicitly measured interpretation biases. Although negative mood and depressive symptoms on the one side and depressive symptoms and negative cognitive schemata on the other side are intercorrelated, we included these variables in order to disentangle the influence of depressive symptoms from a generally negative affective state (including anger, guilt, and anxiety). We additionally explored the complementary influence of state and trait anxiety, given the high comorbidity between depression and anxiety symptoms and the fact that the previous literature has shown increased negative interpretation bias in anxiety disorder. Finally, we assessed the direct convergence of both bias scores by correlating them in a subsample completing both interpretation bias tasks.

## 2. Study 1: Implicit Interpretation Bias

### 2.1. Methods

#### 2.1.1. Participants

Participants were 113 university students (32 male, 81 female) (see Table 1 for sample description) aged between 18 and 36 (M = 21.62; SD = 3.38). The study protocol adhered to the declaration of Helsinki and was approved by the local ethics committee. After a complete description of the study, all participants gave written informed consent before the data collection.

#### 2.1.2. Experimental Task

**Figure 1:**
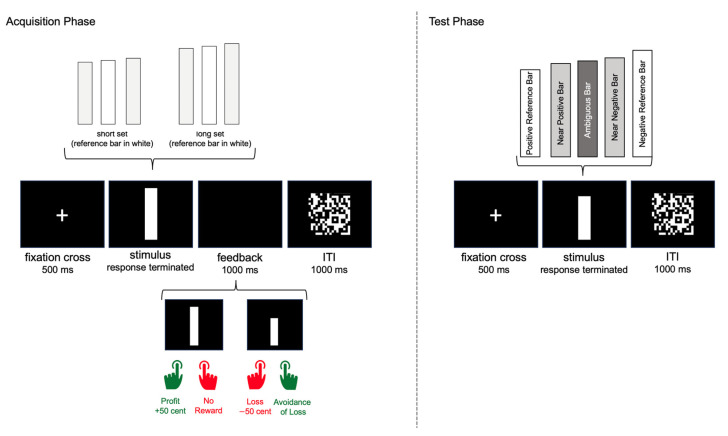
Exemplary trial structures of the acquisition and test phase with the respective timings and stimulus types.

In study 1, we applied the Ambiguous Cue-Conditioning Task, which was programmed and presented with Inquisit 4. We previously adapted this task from an animal experiment [29] to assess implicit interpretation biases in humans [27,28]. In line with the animal work, the original human experiment [27] utilized auditory stimuli of different frequencies as conditioned stimuli. However, for the sake of more practical and straightforward data collection, without the need for an elaborate auditory threshold test and headphone calibration, we employed visual stimuli as conditioned stimuli in the experiment conducted here. More precisely, the visual stimuli were graphical bars of different lengths. In line with the original study protocol, the experiment included two phases: an acquisition and a test phase. Exemplary trial structures of the acquisition and test phase with the respective timings and types of stimuli are depicted in Figure 1.

During the acquisition phase, two different sets of bars were presented: the ‘short set’ consisted of 3 short vertical black bars, where the reference bar had a length of 4.5 cm and the two other bars were −3% shorter (4.37 cm) and +3% longer (4.64 cm). The ‘long set’ consisted of 3 longer vertical bars with the reference bar having a length of 5.5 cm and the two other bars in this set being −3% shorter (5.34 cm) and +3% longer (5.67 cm), respectively. All bars had the same width of 1 cm. The task of participants was to discriminate between the two sets of bars (short vs. long) by pressing one of two response keys. One set of bars is referred to as “positive” as it acquired positive valence over the course of the experiment through monetary gain (profit) after pressing the correct response key. A correct response to the profit set of bars resulted in gaining 50 cents, while pressing the wrong key to this group resulted in no gain (±0 cents). The other set of bars is referred to as “negative” as participants lost money (50 cents) when pressing the wrong response key to this set of bars, while avoiding loss (±0 cents) when pressing the correct response key. Thereby, the two stimulus sets (short and long) were associated either with positive outcome (monetary gain) or potentially negative outcome (monetary loss). The exact procedure and timing for acquisition trials was as follows: Presentation of a fixation cross for 500 ms, followed by one of six vertical black bars presented in random order in the center of a white screen until the participants pressed the right or left response key. After pressing a key, participants received feedback for 1000 ms (e.g., “+50 cents”), followed by a background of black and white pixels for 1000 ms to avoid direct comparison with the length of the next bar (e.g., by an afterimage). The acquisition block consisted of 60 trials, 10 trials per length (6 different bars).

Following the acquisition trials, participants completed the test phase, where only the short and long reference bars (4.5 cm and 5.5 cm) were presented alongside three ambiguous bars, which were of intermediate size. The most intermediate of these ambiguous stimuli was 10% longer than the short reference bar and 10% shorter than the long reference bar (4.95 cm). The other two ambiguous bars were 7% longer than the short reference bar (4.82 cm) and 7% shorter than the long reference bar (5.12 cm). In total, the test phase included 5 distinct stimuli, a positive reference stimulus (PR), a near positive stimulus (NP), an ambiguous stimulus (AMB), a near negative stimulus (NN), and a negative reference stimulus (NR).

In the test phase, participants were asked to sort the presented bars by pressing the left or right key according to the rule detected in the acquisition phase. The procedure was analogous to the acquisition trials, except that the participants received no feedback for their responses. However, they were informed that a counter running in the background of the program would count the responses in each trial and that this counter would also be the basis for calculating the amount of money the participants would receive at the end of the task. The test block consisted of 100 trials, 20 per stimulus type (PR, NP, AMB, NN, NR). In order to rule out systematic effects of bar length and button site, participants were randomly assigned to one of four conditions: (1) Association of the short reference bar and gain with the left button, association of the long reference bar and loss with the right button; (2) Association of the short reference bar and gain with the right button, association of the long reference bar and loss with the left button; (3) Association of the reference short bar and loss with the left button, association of the long reference bar and gain with the right button; (4) Association of the short reference bar and loss with the right button, association of the long reference bar and gain with the left button. Finally, to assess the association of bar length with profit or loss, we asked participants to rate the reference bars on the Self-Assessment Manikin scale for valence [35] ranging from 1 = “very unpleasant” to 9 = “very pleasant”. The presentation order of the two bars (shorter and longer reference bars) in this rating task was randomized.

The experiment was programmed in such a way that all participants won 5 €, which study participants received after completion of the study.

#### 2.1.3. Procedure and Data Assessment

Participants completed German versions of the following questionnaires before the experimental test session: the Dysfunctional Attitude Scale [36] to assess dysfunctional attitudes and cognitive schemata, the State-Trait Anxiety Inventory [37] to measure trait and state anxiety, and the Beck Depression Inventory-II (BDI-II; [38]) to measure levels of depression. Immediately before running the ACCT paradigm, participants completed the Positive and Negative Affect Schedule [39] to assess current positive and negative mood. For all scales, reliability indices (Cronbach’s alpha) for the sample of Study 1 were all satisfactory to very good (DAS α = 0.88; STAI-T α = 0.92; BDI-II α = 0.89; STAI-S α = 0.86; PANAS positive α = 0.74; PANAS negative = 0.68).

#### 2.1.4. Data Reduction and Analysis

To analyze learning behavior during the acquisition phase, we summed up correct responses to the presented bars (3 bars associated with gain, 3 bars associated with potential loss) and divided this number by the total number of presented bars of that category. This resulted in a score of 1 for 100% correct responses to 0 for no correct response. Only participants with at least 55% of correct answers to the respective bars in the acquisition phase were included in the analysis, which were all participants in the present study.

For the analysis of the interpretation bias, we analyzed the testing phase. We calculated a score for each stimulus category (PR, NP, AMB, NN, NR) by coding positive response button presses (i.e., the key associated with the positive reference bar) with 1 and negative response button presses (i.e., the key associated with the negative reference bar) with −1, summing up these coded values, and dividing them by the total number of trials per stimulus category. This resulted in a score ranging from −1 (only negative answers) to 1 (only positive answers).

Statistical data analyses were carried out using SPSS Statistics [40]. Descriptive statistics for the mean correct responses to the presented bars in the acquisition phase were calculated. Further, we calculated two repeated-measures analyses of variance (rmANOVA) for data of the test phase: (1) One, including the bias score for all stimuli presented during the test phase as repeated measures factor, resulting in a five-stage factor (PR, NP, AMB, NN, NR); and (2) the second with SAM scores for the positive and negative reference bars, respectively, as dependent variables and valence (PR, NR) as repeated-measures factor. The first rmANOVA served as a manipulation check with the aim of determining the expected differences in responses to the different stimuli representing a linear function from the positive reference stimuli to the negative reference stimuli. Thus, a main effect of the type of bar was expected here. The second rmANOVA tested the hypothesis that positive and negative reference stimuli acquired different valence values during the acquisition phase, supposed to result in a significant main effect of valence. Finally, a hierarchical multiple linear regression analysis was conducted with the score relating to the middle (ambiguous) bar as bias score and criterion (dependent variable). The other stimuli (bars) were not included in this regression analysis. With this hierarchical regression analysis, we investigated which depression-related state and trait variables predict the variance in the implicit bias score (from the ACCT) beyond that already accounted for by age and sex and whether state- and trait-anxiety additional adds to variance explanation in the bias score. To this end, forced entry was used for the first block of predictors, comprising age and sex. The second block of predictors included depressive symptoms (BDI), negative mood (PANAS), and dysfunctional cognitive schemata (DAS), because our main hypothesis was that a negative interpretation bias is predicted by depression-related state and trait characteristics. The third block included state and trait anxiety variables that might influence the negative interpretation bias above and beyond depression-related states and dysfunctional schemata. Finally, we included a fourth block into the regression model, containing the subjective evaluation of the reference stimuli’s valence, as we suggested that the extent to which the reference stimuli are evaluated positively or negatively might influence the degree of a negative interpretation bias. As previous empirical findings and theoretical considerations did not allow an a priori selection of the predictors, we used a stepwise procedure within the second, third, and fourth block of variables rather than forced entry. Regression diagnostics were performed to test for collinearity, normality, outliers, and leverage. In line with Urban and Mayerl [41], we considered a tolerance score of >0.25 and a VIF score <5.0 as collinearity criteria for the regression analysis. Finally, we calculated Pearson correlation coefficients between the trait and state predictor variables and the criterion.

### 2.2. Results

**Figure 2:**
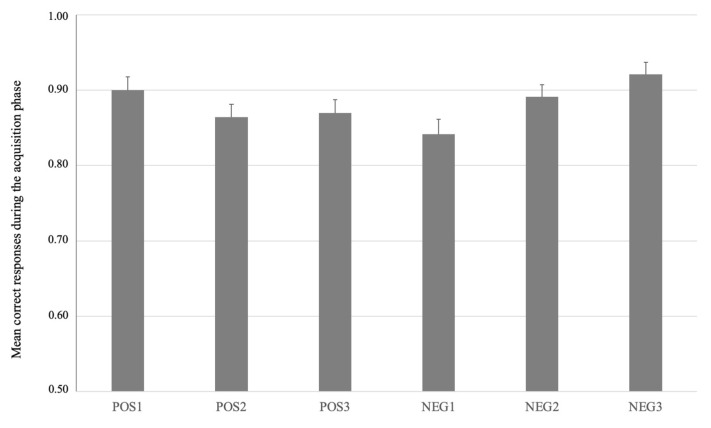
Mean correct responses (with standard error bars) for the acquisition phase for the three vertical bars associated with gain (POS1, POS2-R, POS3) and the three vertical bars associated with potential loss (NEG1, NEG2-R, NEG3). Bars in dark grey (POS2-R and NEG2-R) were used as reference bars during the test phase.

Analysis of the acquisition phase showed a high mean correct response rate for the two reference stimuli ranging between 0.86 and 0.94 (see Figure 2). These response rates show that individuals learned the association between the respective bars and response button correctly.

The rmANOVA of the mean positive response button presses (button associated with the positive reference bar) to the five different bars during the test phase revealed a significant main effect (F(1,113) = 836.24; *p* < 0.001; η^2^ = 0.88), indicating a linear degrade of positive response button presses from the positive to the negative reference bar. The rmANOVA of the subjective valence of the positive and negative reference bars showed a significant main effect (F(1,113) = 17.95; *p* < 0.001; η^2^ = 0.13), indicating more positive evaluation of the positive reference bar as compared to the negative reference bar (Table 2).

For the hierarchical regression analysis to identify significant predictors of the implicit interpretation bias as assessed with the ACCQ, the first model, including age and sex in block one, was not significant in predicting the mean implicit interpretation bias in the ACCT paradigm (Model 1; see Table 3). This model accounted for 3% (R-adjusted 1%) of the variance in the interpretation bias. From block two (depression-related variables: depressive symptoms, negative mood, dysfunctional schemata), block three (state and trait anxiety), and block four (subjective valence of the reference bars), none of the predictors survived the significance threshold so that no further model significantly improved the prediction of the interpretation bias. Pearson correlation coefficients between the implicit bias score (from the ACCT) and the psychological predictor variables were all low and not significant (see Table 4; for the detailed statistical indices of the variables excluded from the respective regression models see Table S1 in the Supplementary Materials).

## 3. Study 2: Explicit Interpretation Bias

### 3.1. Methods

#### 3.1.1. Participants

Participants were 113 university students (24 male, 89 female) (see Table 1 for sample description) aged between 22 and 45 (M = 25.74; SD = 4.14). The study protocol adhered to the Declaration of Helsinki and was approved by the local ethics committee. After a complete description of the study, all participants gave written informed consent before data collection.

#### 3.1.2. Experimental Task

##### Development of Stimulus Material

The DUCTUS paradigm was developed as an ambiguous social scenarios test with description of interpersonal situations as main stimulus material. The aim was to select social situations that enabled the assessment of one of several types of cognitive errors. To this end, we translated N = 162 social scenarios from previous studies [42,43] into German using a professional translator. Two clinical psychologists independently categorized all scenes with respect to the type of cognitive error. Over all scenes, Cohen’s Kappa was significant (α < 0.001) with an interrater correlation of 0.45 which is acceptable [44]. By selecting those scenes for which both raters had chosen the same type of cognitive error, we ended up with 80 short descriptions of interpersonal situations. The scenario descriptions were adjusted to halt at a point that left the outcome of the situation open-ended, i.e., the situation at that point could have a positive, negative, or neutral outcome (e.g., “While you are shopping you notice a friend who you haven’t seen in several years. You walk up to him to say ‘hi’ but he walks past you. As you catch up to him and introduce yourself you think that….”). Subsequently, three interpretation options were formulated for each scene: a negative (e.g., “maybe he ignored you on purpose”), a positive (e.g., “he will be very excited about meeting you”), or a neutral (e.g., “he just didn’t recognize you”) interpretation of the situation. The negative option represented one of four types of cognitive errors (disqualifying the positive n = 17, catastrophizing n = 22, black-and-white thinking n = 19, jumping to conclusions n = 22) to mimic cognitive biases that are typical for a depressive thinking style. Positive interpretations were not chosen to represent a particular cognitive distortion (e.g., unrealistic optimism, positive overgeneralization). A neutral option was included as a distractor and because previous studies [43] have indicated that the lack of a neutral alternative could lead to over- or underestimation of effects. Furthermore, for 27 scenes from the item pool that were initially not selected according to the above-mentioned criterion, we designed only neutral interpretations (i.e., three neutral response options) to reduce the risk of systematic response tendencies.

##### Experimental Design

The final DUCTUS paradigm includes 80 short descriptions of interpersonal situations that are ambiguous in appraisal with a positive, negative, and neutral interpretations (response options) and 27 scenes with only neutral response options. Each subject completed a randomized sequence of 40 items of the stimulus pool of 80 ambiguous stories as well as 14 out of 27 neutral scenes as distractor items.

**Figure 3:**
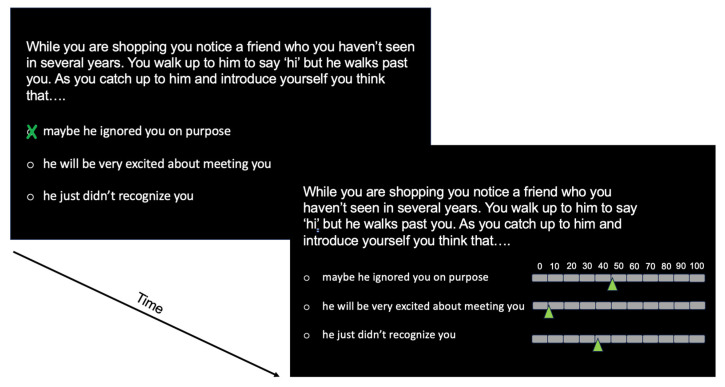
Exemplary trial structures of the DUCTUS-T.

During the experiment, participants were first presented with the description of the social situation and the three response options (positive, negative, neutral for the ambiguous scenarios of interest and only neutral for the distractor items). They were asked to empathize with the situation and to choose the interpretation that they believe best represents the thoughts they would have, facing the specific situation (see Figure 3 for a graphical depiction of an exemplary trial of the DUCTUS paradigm). After response selection a second screen appeared, asking the participants to assign a probability between 0–100% to each of the three options for interpretation by stating how likely it was that they would have interpreted the situation according to the chosen thought (0% = I would certainly not have thought this, 100% = I would certainly have thought this). The analysis of the second inquiry is not included in this paper, as we wanted to keep the bias scores of the two experiments as similar as possible in terms of the instantaneous interpretation and reaction to a given stimulus.

#### 3.1.3. Procedure and Data Assessment

As in Study 1, participants completed the German versions of the following questionnaires right before conducting the experiment: the Dysfunctional Attitude Scale (DAS-40) [36] to assess dysfunctional attitudes similar to negative cognitive schemata—for the DAS-40 the overall sum score was used; the State-Trait Anxiety Inventory (STAI) [37] to measure trait and state anxiety; and the WHO-FIVE Well-being Index (WHO-5) [45] to measure levels of depression. Immediately before running the DUCTUS paradigm, participants completed the Positive and Negative Affect Schedule (PANAS) [39] to control for positive and negative affective states. The questionnaires used do not necessarily reflect clinical dysfunction or diagnoses; however, as we investigated a sample of healthy individuals we were mainly interested in determining continuous levels of the respective constructs.

For all scales, reliability indices (Cronbach’s alpha) for the sample of Study 2 were all satisfactory to very good (DAS α = 0.88; STAI-T α = 0.90; WHO-5 α = 0.84; STAI-S α = 0.89; PANAS positive α = 0.82; PANAS negative = 0.74).

Finally, after completion of the DUCTUS paradigm they filled out the Cognitive Error Questionnaire [46], which includes a total score as well as subscales representing four types of cognitive errors (catastrophizing, overgeneralization, personalization, selective abstraction). Lower scores on the CEQ indicate higher tendency for cognitive errors. The German version of the CEQ has internal reliabilities ranging from satisfactory to very good (Cronbach’s α = 0.59–0.87). The CEQ was included to evaluate construct validity of the newly developed DUCTUS paradigm. The total score of the CEQ significantly correlated with the mean bias score of the DUCTUS paradigm to 0.47 (*p* < 0.001).

#### 3.1.4. Data Reduction and Statistical Analysis

For data reduction purposes, the initial choice of an interpretation alternative was coded with −1 for the negative interpretation, with 0 for the neutral interpretation and +1 for the positive interpretation for those 40 stories that included positive, neutral, and negative interpretations. For all these social situations and thus answers of an individual, we computed a mean of these recoded values, resulting in an overall bias score that could range between −1 for a complete negative bias and +1 for a complete positive bias. Distractor stories with neutral interpretations only were discarded from further analyses.

All statistical data analyses were carried out using SPSS Statistics Version 23 [40]. As in study 1, we calculated a hierarchical multiple linear regression analyses, this time with the mean bias score (ranging between −1 for a complete negative bias and 1 for a complete positive bias) calculated from the forced choice answers as dependent variable. The regression analysis was set up identical as in Study 1 except for the fourth block (valence ratings of reference tones in the ACCT), which was not available for the analysis of the DUCTUS bias score. Again, regression diagnostics were performed to test for collinearity, normality, outliers, and leverage. In line with Urban and Mayerl [41], we considered a tolerance score of >0.25 and a VIF score <5.0 as collinearity criteria for the regression analysis. Finally, we calculated Pearson correlation coefficients between the trait and state predictor variables and the criterion.

To investigate the relationship between both bias scores, we calculated a Pearson correlation coefficient between the interpretation bias scores from the ACCT and the DUCTUS-T for the subsample of Study 2 completing both tasks.

### 3.2. Results

Means and standard deviations for the explicit bias scores as measured with the DUCTUS-T are displayed in Table 2.

The hierarchical regression analysis, for which the statistical indices of the significant models and predictors are displayed in Table 4, revealed the following results: Age and sex, entered in block one, significantly contributed to predicting the mean explicit interpretation bias in the DUCTUS paradigm, but only sex was a significant predictor (with females showing a more negative bias than males), whereas age was not (Model 1). From block two (depression-related measures), the variable ‘dysfunctional cognitive schemata (DAS)’, was included in the model in a first step, significantly improving the prediction of mean explicit interpretation bias (Model 2) beyond age and sex. With the addition of the variable depressive symptoms (WHO-5, also from block two), the prediction of the explicit interpretation bias was further improved (Model 3). No further variables from block two (negative mood) or block three (state and trait anxiety) were included as significant predictors of the explicit interpretation bias, so that the resulting final model (Model 3) included the predictors sex, dysfunctional attitudes, and depressive symptoms, accounting for 40% (R-adjusted 38%) of the variance in the explicit interpretation bias from the DUCTUS-T. Pearson correlation coefficients between the explicit bias score from the DUCTUS-T and the psychological predictors variables were all significant except negative mood (see Table 5; for the detailed statistical indices of the variables excluded from the respective regression models see Table S2 in the Supplementary Materials).

The correlational analyses in the subsample completing both the explicit bias (DUCTUS-T) and the implicit interpretation bias (ACCT; N = 46) revealed a low and non-significant correlation between the implicitly (ACCT) and explicitly (DUCTUS) measured interpretation bias (r = −0.10; *p* = 0.50).

## 4. Discussion

The present study aimed at investigating differential predictors of implicitly and explicitly measured depression-related interpretation bias in a sample of young, healthy individuals. Among the tested predictors, sex, dysfunctional schemata, and depressive symptoms significantly predicted an explicitly measured negative interpretation bias with a substantial variance explanation of 40%. None of the included demographic nor depression- or anxiety-related state and trait characteristics significantly predicted the implicitly measured interpretation bias (ACCT), resulting in a very low proportion of explained variance (3%). Furthermore, the implicitly and explicitly measured interpretation bias showed only a low and non-significant relationship. These results reveal a dissociation between implicitly and explicitly measured interpretation biases as well as their respective predictors and raise several important questions that are discussed below.

The results of our explicit measure of the Interpretation bias support cognitive theories, proposing that a negative interpretation of ambiguous information in individuals with depressive symptoms results from negative cognitive schemata that trigger mood-congruent semantic information which is used to interpret a given situation [3,4]. In our study, such theoretical accounts are backed by the significant prediction of a negative interpretation bias through dysfunctional cognitive schemata and depression symptoms as measured by the short screening measure WHO-5. Our results are in line with previous research in patients with unipolar depression, reporting a negative bias in the completion of open sentences (e.g., [47]), the scrambled sentence task [48,49,50], or in the evaluation of ambiguous scenarios (e.g., [51,52]). Further, significant correlations have also been shown in nonclinical individuals scoring high on a depression inventory [53] and, in medical students, a positive interpretation bias at baseline predicted higher trait resilience and lower depressive symptoms 6 months later [54]. Although previous studies have linked negative interpretation bias to anxiety symptoms (e.g., social phobia, generalized anxiety disorder, posttraumatic stress disorder [16]) our results do not show an additional prediction of interpretation bias by trait or state anxiety over and above the depression-related variables. However, simple correlations between trait and state anxiety and the DUCTUS-T interpretation bias were significant, indicating higher anxiety levels in relation to negative interpretation bias (STAI-T: r = −0.34; *p* < 0.001; STAI-S: r = −0.32; *p* < 0.001). Several reasons could account for this finding: first, anxiety disorders often co-occur with comorbid depressive symptoms [55] and vice versa [56], rendering it difficult to disentangle the differential effects of anxiety and depression on interpretation biases (e.g., [57]). Second, there is strong evidence for a biased allocation or targeting of attention towards even subliminal (or masked) presented threatening stimuli, indicating an altered pre-conscious information processing for anxiety [58]. In contrast, depression might be more related to post-attentional, higher-order processing biases, as there is clear evidence of interpretation and memory biases in depression but inconclusive for anxiety [59,60], indicating a possible disorder-specific disturbed information processing pattern along the emotion-generation stream. Third, from a methodological perspective, the superiority of dysfunctional attitudes and depression in predicting negative interpretation bias in our study might also be related to the type of potential interpretation the individual had to choose with respect to the ambiguous scenes. The negative interpretation alternative was designed to represent socially/interpersonal-relevant, depressive rather than threat-related, anxious cognitive distortions. This may be important, in that meta-analyses within the cognitive bias literature have already established a content-specific superiority of attentional biases for disorder-congruent, compared to more global, disorder-irrelevant threatening stimuli [61]. Further, the measure of anxiety used in the present study was very general, assessing general trait anxiety with no specific relationship to an anxiety disorder, whereas previous studies, reporting stronger negative interpretation bias in anxious individuals, have used more specific questionnaires for social anxiety or generalized anxiety disorder (e.g., [62,63,64]).

We identified sex as significant predictor of the explicit interpretation bias, which fits well with the previous literature demonstrating a greater negative interpretation bias for ambiguous information in females than in males (e.g., social scenes [65]; neutral facial expressions [66]). Such a negative cognitive style exhibited by girls/women may pose a putative risk factor leaving them more vulnerable to emotional disorders, as indicated by an epidemiological, almost twice as likely risk for woman than men to develop a depressive or anxious psychopathology during their lifetime [67]. In contrast to earlier reports revealing an age-related positivity bias for cognitive and emotional information processing in older age (e.g., [68,69]), age did not contribute substantially to the variance explanation of the implicit and explicit interpretation bias scores in our study, which might also be explained by the relatively small variance in age (study1: 22–45 years; study 2: 18–36 years).

Apart from the described relationships between dysfunctional schemata and depressive symptoms on the one side and the negative interpretation bias on the other, it has to be noted that overall study participants in the present study exhibited a positive interpretation bias, which is in line with previous studies (e.g., [70,71]). Our regression and correlational results might therefore support the hypothesis that with an increase in depressive symptoms, and in the present study also with more dysfunctional attitudes, a positive bias erodes and a negative bias begins to evolve [71,72].

Whereas the present study, in line with previous studies, reports quite consistently a significant relationship between depressive symptoms and dysfunctional schemata and an explicitly measured negative interpretation bias, none of the predictors included in the regression model (dysfunctional schemata, trait and state anxiety, depression, and current negative mood) reached statistical significance for the implicitly measured bias, nor did the model with the entered predictors age and sex reach statistical significance. These null findings are backed up by a lack of significant correlations between the predictors and the interpretation bias assessed via the ambiguous cue conditioning task. Furthermore, both bias scores from the ACCT and the DUCTUS task did not significantly correlate with each other. Our results are partly in line with previous studies, using performance-based and thus implicit measures of the interpretation bias. For example, Lawson and MacLeod [24] failed to find evidence for a relationship between depression scores and negative bias in an affective priming task, but the same research group could observe a negative bias, as reflected by larger startle reflex amplitudes to ambiguous words in individuals with higher BDI scores [25].

Several lines of argumentation might explain our results for the implicit interpretation bias and the discrepancies between explicit and implicit bias measures. First, the lack of correlations could reflect a methodological, in this case a measurement, problem due to different assessment methods. Whereas in Study 1 the predictors and the criterion were both measured explicitly (i.e., questionnaires, DUCTUS paradigm), Study 2 employed predictors that were assessed on a self-report level, whereas the criterion was measured with an implicit paradigm. Although a valid construct should be measurable by different approaches (e.g., self-report, observation, behavior) as proposed by Campbell and Fiske [73], studies using the multi-trait—multi-method matrix rather suggest the existence of several independent components of the measured trait instead of one uniform trait [74]. In this vein, the field of attitude research, mainly rooted in personality and social psychology, has raised the assumption that explicit and implicit measures of attitudes provide different aspects of the attitude that might even conflict with each other (e.g., [75]) but nevertheless both contribute to a certain behavior (see also [76]). Indeed, with respect to the present study, the interpretation biases measured with the DUCTUS-T and the ACCT might represent different processes and therefore different aspects of the overall construct of an interpretation bias. In the DUCTUS-T, individuals are supposed to appraise the social situation in a certain way and, depending on their cognitive schemata and affective state, consciously select one of the proposed alternatives as their own potential interpretation of the situation. In contrast, the bias score in the ACCT represents an automatic interpretation of the ambiguous stimulus, representing an implicitly learned tendency to approach reward or avoid punishment rather than a conscious appraisal of the given situation. However, this remains speculative, unless, for example, neuroimaging studies have clarified the underlying neural activation patterns that mediate implicit and explicit interpretation biases as operationalized in the present study.

Second, differences in the self-relevance of the presented stimuli could be relevant. Whereas in the DUCTUS paradigm we asked participants to select the interpretation that comes nearest to the thought they would have in the situation, the ACCT did not require any retrieval of self-relevant information. Some authors have shown that negative interpretation of, e.g., social situations is more pronounced when depressed individuals imagine themselves being part of the situation as compared to imagining others [77]. In the same vein, depressed individuals in comparison to non-depressed individuals are more pessimistic when imagining self-relevant future events [78,79,80].

Finally, our differential results for explicitly and implicitly measured bias scores could also reflect two different mechanisms resembling two different mathematical functions: first, for the explicitly measured bias a variation in the tendency to interpret ambiguous situations more negatively might covary linearly with the variation in trait and state characteristics on a continuum from healthy to psychopathological (see erosion hypothesis above); second, and in contrast, the implicitly measured bias might rather resemble an exponential function in which the automatic choice of approach or avoidance behavior (i.e., negative bias) does not change until a certain level of trait and state characteristics exceeds a pathological threshold. This hypothesis is very speculative and needs to be confirmed in future studies directly comparing explicit and implicit measures of the interpretation bias in a large sample covering a wide range of depressive (and anxiety) symptoms from healthy individuals over subsyndromal up to clinical depression.

The results of the present study must be considered in the light of some limitations. First, we only investigated a relatively small subsample of participants performing on both experimental paradigms in order to directly compare the shared and different mechanisms of explicit and implicit interpretation biases. Unfortunately, the sample size was too small to perform hierarchical regression analyses in this subsample.

Second, the present study used a cross-sectional design that did not allow us to identify differential causal contributions of each of the investigated state or trait variable to a negative interpretation bias. This holds particularly true as constructs used in the present study to predict interpretation biases are inter-correlated. Therefore, future longitudinal studies in large diverse samples are needed to confirm the differential contributions of depressive symptoms, negative mood, and negative cognitive schemata to negative interpretation biases.

Third, our results from the first study partly contradict previous results from our group using a previous version of the ACCT [27,28] which reported significant correlations, with one aspect of rumination (reflective pondering) in one study [27] and anxiety in the other [28]. However, in these studies we investigated a much smaller sample of N = 20 and N = 25, respectively, and might therefore have overestimated effects. Further, we used a slightly different experimental setup in these studies, with tones instead of graphical bars as reference and ambiguous stimuli. In our view, there is, however, no reason to argue that this difference in stimulus modality systematically affected the results. Accordingly, previous studies showed that the acquisition of conditioned responses are comparable between auditory and visual conditioning stimuli (e.g., [81]).

Fourth, the two studies did not use identical questionnaires for depression symptoms. Whereas Study 1 utilized the BDI-II (21 items), Study 2 employed the WHO-5 screening instrument with only five items. This decision was made to reduce the number of questionnaire items, as additional psychological constructs, not relevant to this article, were assessed through self-report in Study 2. Opting for the WHO-5 also presented the drawback that it is a well-being screening instrument and not a diagnosis-specific tool for depression. However, the WHO-5 demonstrates high correlations with questionnaires measuring current depressive symptoms (e.g., [82]). Additionally, a systematic review [83] indicates a high clinimetric validity of the WHO-5 index, which serves as a sensitive and specific screening tool for depression with extensive applicability in research. Furthermore, although the WHO-5 was originally designed to measure well-being, empirical findings suggest its effective use in the context of depression research. A study by Krieger and colleagues [84] demonstrated that the WHO-5 index is highly negatively correlated with self-reports and observer-ratings of depressive symptoms, particularly in the realm of mild and moderate depression symptoms, as commonly observed in healthy samples such as the present one. In the mentioned study by Krieger et al., these correlations remained substantial even when controlling for anxiety symptoms. Nevertheless, future studies should assess both interpretation biases (implicit and explicit) in the same sample, using identical psychometric instruments for depressive symptoms.

Although the present study investigated healthy individuals, the results suggest some clinical implications that should be investigated further in future studies. Our findings for the explicitly assessed bias linked to depressive symptoms align with current etiological models of depression development and maintenance, suggesting that addressing conscious cognitions may offer promise in treating depressive symptoms. Furthermore, training healthy distressed individuals (with more depressive symptoms, negative affect, and dysfunctional cognitive schemas) in interpreting ambiguous situations more positively—according to cognitive bias modification methods—might increase their resilience. In contrast, results from implicit procedures exhibit more inconsistencies, with outcomes being contingent on the specific implicit task and the severity of depression [85]. From our results and others, it seems plausible that more automatic, unconscious dysfunctional cognitive processes are only discernible in clinical depression. For instance, the Implicit Association Test identified low implicit self-esteem in patients with depression but not in individuals with remitted depression [86]. If this holds true in future studies with larger and clinical samples, it would enable us to use an implicit bias, as assessed with, e.g., the ACCT, to detect clinical depression in an early stage or potentially also to predict treatment response. However, these implications are still very speculative and need more specific examination in the future.

## 5. Conclusions

For explicitly measured negative interpretation biases, the present studies revealed empirical evidence for theories suggesting a vicious circle of negative schemata and depression symptoms. In contrast to the explicitly measured bias but in line with previous studies, we were not able to show significant relationships between an implicitly measured negative interpretation bias and any of the above-mentioned predictors, nor did we observe a significant correlation between the different bias scores. These divergent results support the hypothesis that a negative interpretation bias might be comprised of different aspects that are not necessarily in straight correspondence: a relatively automatic approach to or avoidance of a stimulus/situation due to implicitly learned associations (implicit bias) and a conscious appraisal of a certain situation (explicit bias). The divergent results might therefore not signify invalidity of one or the other measure but rather underline the multidimensionality of a construct [75,76]. This provides valuable information, as the different dimensions might be expressed differentially in healthy individuals with subsyndromal depressive symptoms and in patients with major depression.

## Figures and Tables

**Table 1:** Means and standard deviations of demographic characteristics as well as trait and state variables entered into the hierarchical regression analyses.

	Study 1 (N = 113)	Study 2 (N = 113)	Study 2 Subsample (N = 46)
	Mean (SD) Range	Mean (SD) Range	Mean (SD) Range
Age	21.62 (3.38) 18–36	25.74 (4.14) 22–45	25.97 (3.67) 22–39
Sex (N; female/male)	81/32	89/24	26/20
WHO	---	15.83 (4.21) 5–25	16.09 (3.67) 5–22
BDI-II	4.60 (5.43) 0–44	---	---
PANAS negative mood	1.24 (0.45) 1–5	1.63 (0.49) 1–3	2.05 (0.32) 1–3
DAS Total	115.42 (24.53) 71–180	115.09 (22.66) 66–173	114.14 (21.60) 75–163
STAI Trait	36.83 (10.01) 22–74	34.67 (8.50) 21–66	36.16 (9.01) 21–66
STAI State	36.94 (7.77) 20–56	38.38 (4.30) 24–57	39.14 (7.87) 24–56

Abbreviations: WHO-5 = WHO-FIVE Well-being Index; BDI-II = Beck Depression Inventory-II; PANAS = Positive and Negative Affect Schedule; DAS = Dysfunctional Attitudes Scale; STAI = State-Trait Anxiety Inventory.

**Table 2:** Means and standard deviations of experimental measures of Study 1 (ACCT) and Study 2 (DUCTUS) and the subsample of Study 2 completing both tasks.

	**Study 1: ACCT**			
	**Total Sample (N = 113)**	**Female (N = 81)**	**Male (N = 32)**	**Subsample Study 2 (N = 46)**
	**Mean (SD)**	**Mean (SD)**	**Mean (SD)**	**Mean (SD)**
Positive reference cue (PR)	0.88 (0.21)	0.86 (0.23)	0.94 (0.11)	0.86 (0.27)
Near positive cue (NP)	0.29 (0.47)	0.23 (0.49)	0.43 (0.39)	0.25 (0.53)
Ambiguous cue (AMB)	−0.13 (0.44)	−0.17 (0.44)	−0.02 (0.44)	−0.11 (0.57)
Near negative cue (NN)	−0.49 (0.42)	−0.52 (0.39)	−0.42 (0.48)	−0.49 (0.47)
Negative reference cue (NR)	−0.92 (0.17)	−0.92 (0.18)	−0.93 (0.14)	−0.92 (0.20)
Valence positive cue *	5.86 (1.73)	5.60 (1.49)	6.62 (1.83)	5.47 (1.45)
Valence negative cue *	4.96 (1.45)	4.90 (1.39)	5.09 (1.61)	4.91 (1.34)
	**Study 2: DUCTUS**			
	**Total Sample (N = 113)**	**Female (N = 89)**	**Male (N = 24)**	**Subsample Study 2 (N = 46)**
	**Mean (SD)**	**Mean (SD)**	**Mean (SD)**	**Mean (SD)**
Disqualifying the positive	0.23 (0.25)	0.21 (0.26)	0.32 (0.21)	0.24 (0.27)
Catastrophizing	0.24 (0.28)	0.20 (0.29)	0.39 (0.19)	0.23 (0.31)
Black and white thinking	0.21 (0.25)	0.19 (0.27)	0.27 (0.20)	0.26 (0.20)
Jumping to conclusions	0.07 (0.30)	0.03 (0.32)	0.20 (0.24)	0.13 (0.28)
Total	0.18 (0.19)	0.15 (0.20)	0.29 (0.13)	0.19 (0.17)

* As measured with the Self-Assessment Manikin ranging from 1 (=negative) to 9 (=positive).

**Table 3:** Stepwise hierarchical regression analysis predicting the mean bias score in the ACCT paradigm (N = 113). Statistics for the significant models and for the variables included in the respective models (see Supplementary Materials for indices of the variables excluded from the regression models).

	β	T	*p*	R^2^	R^2^ Adj	F (Model)	*p* (Model)
Model 1							
Block 1: Age	−0.01	−1.08	0.28				
Block 1: Sex	0.08	1.63	0.11				
Model Statistics				0.03	0.01	1.71	0.19

Abbreviations: Adj, adjusted; β, standardized beta coefficient.

**Table 4:** Pearson correlation coefficients between the mean bias score from the ACCT paradigm and the mean bias score from the DUCTUS-T and the respective predictor variables included in the hierarchical regression analyses (N = 113).

	Bias Score ACCT	Bias Score DUCTUS
DAS	0.071	−0.482 **
STAI-T	−0.107	−0.343 **
STAI-S	−0.041	−0.320 **
BDI/WHO-5 ^†^	0.015	0.423 **
PANAS negative	−0.139	−0.037
Valence PR	−0.023	---
Valence NR	0.022	---

** = *p* < 0.01; significant correlations are shown in bold; DAS = Dysfunctional attitude Scale; STAI_T = State-Trait Anxiety Inventory-Trait Version; STAI_S = State-Trait Anxiety Inventory-State Version; WHO-5 = WHO-FIVE Well-Being Index; PANAS negative = Positive and Negative Affect Schedule-negative affect; **^†^** BDI for Study 1 and WHO-5 for Study 2.

**Table 5:** Stepwise hierarchical regression analysis predicting the mean explicit bias score in the ACCT paradigm (N = 113). Statistics for the significant models and for the variables included in the respective models. Statistical indices of the variables excluded from the respective regression models are depicted in the Online Supplementary Materials File.

	β	T	*p*	R^2^	R^2^ Adj	F (Model)	*p* (Model)
Model 1							
Block 1: Age	−0.09	−0.93	0.36				
Block 1: Sex	−0.33	−3.46	<0.001				
Model Statistics				**0.11**	**0.09**	**6.06**	**0.003**
Model 2							
Block 1: Age	−0.07	−0.85	0.39				
Block 1: Sex	−0.32	−3.78	<0.001				
Block 2: DAS	−0.47	−5.69	<0.001				
Model Statistics				**0.33**	**0.31**	**32.36**	**<0.001**
Model 3							
Block 1: Age	−0.002	−0.03	0.98				
Block 1: Sex	−0.27	−3.28	0.001				
Block 2: DAS	−0.42	−5.18	<0.001				
Block 3: WHO-5	−0.28	3.37	0.001				
Model Statistics				**0.40**	**0.38**	**11.35**	**0.001**

Abbreviations: Adj, adjusted; β, standardized beta coefficient; significant results are shown in bold.

## Data Availability

The data presented in this study are available on request from the corresponding author.

## Supplementary Materials

The following supporting information can be downloaded at: https://www.mdpi.com/article/10.3390/brainsci13121620/s1, Table S1: Detailed statistical indices for the hierarchical regression Study 1 (ACCT); Table S2: Detailed statistical indices for the hierarchical regression Study 2 (DUCTUS-T).
