# Supplementary material for: Dissociation of Implicit and Explicit Interpretation Bias: The Role of Depressive Symptoms and Negative Cognitive Schemata
Source: Brain Sci. 2023 Nov 22;13(12):1620. doi: 10.3390/brainsci13121620 (PMC10742311; doi:10.3390/brainsci13121620)
Supplement: Supplementary file 1 [file brainsci-13-01620-s001.zip › brainsci-2649169-supplementary.pdf]

Table S1. Detailed statistical indices of the variables excluded from the respective regression models during the stepwise hierarchical regression analysis predicting the mean bias score in the cue conditioning paradigm (N = 113) from trait and state affective variables.

	β	T	p (predictor)
**Model 1**			
BDI	.017	.174	.862
PANAS Negativ	-.116	-1.188	.238
STAI-S	-.003	-.031	.976
DAS	.083	.853	.395
STAI-T	-.072	-.716	.475
Valence PR	-.041	-.431	.680
Valence NR	.000	-.005	.996

Abbreviations: β, standardized beta coefficient

Table S2. Detailed statistical indices of the variables excluded from the respective regression models during the stepwise hierarchical regression analysis predicting the mean bias score in the DUCTUS paradigm (N = 113) from trait and state affective variables.

	β	T	p (predictor)
**Model 1**			
WHO-5	.370	4.014	<.001
PANAS Negativ	-.084	-.887	.377
DAS	-.471	-5.689	<.001
STAI-S	-.255	-2.694	.008
STAI-T	-.315	-3.370	.001
**Model 2**			
WHO-5	2.82	3.370	.001
PANAS Negativ	-0.41	-.491	.625
STAI-S	-.195	-2.319	.022
STAI-T	-.177	-1.995	.049
**Model 3**			
PANAS Negativ	-.062	-.780	.438
STAI-S	-.129	-1.534	.128
STAI-T	-.052	-.527	.600

Abbreviations: β, standardized beta coefficient
